# Psychological Capital and Occupational Well-Being: Mediating Effects of Work Engagement Among Chinese Special Education Teachers

**DOI:** 10.3389/fpsyg.2022.847882

**Published:** 2022-04-05

**Authors:** Qiang Guo, Yongli Wang, Qiaoyun Liu, Tingzhao Wang, Lei Zhang, Zhijun Huang, Shuqin Cao

**Affiliations:** ^1^Hangzhou College of Preschool Education, Zhejiang Normal University, Hangzhou, China; ^2^Department of Rehabilitation Sciences, Faculty of Education, East China Normal University, Shanghai, China; ^3^School of Education, Shaanxi Normal University, Xi’an, China; ^4^Changchun Normal College, Changchun, China; ^5^Department of Special Education, Faculty of Education, East China Normal University, Shanghai, China

**Keywords:** psychological capital, occupational well-being, work engagement, special education teachers, absorption, dedication

## Abstract

This study examines whether psychological capital (PsyCap) indirectly predicts occupational well-being among Chinese special education teachers through work engagement. In total, 615 Chinese special education teachers (female = 567) completed the Psychological Capital Questionnaire, the Special Education Teachers’ Occupational Well-Being Questionnaire, and the Utrecht Work Engagement Scale. The results indicated that PsyCap was positively correlated with occupational well-being and work engagement. Furthermore, work engagement mediated the influence of PsyCap on occupational well-being. Notably, the multiple mediation model indicated that the indirect effects of PsyCap on occupational well-being were mainly due to dedication and absorption. The study’s results illustrate the association between PsyCap, work engagement, and occupational well-being, which may help educational administrators and social workers assist with special education and special education teachers develop and maintain good working conditions.

## Introduction

One of the key factors affecting the development of special education is the quantity and quality of teachers (Billingsley, 2004). Unfortunately, the turnover rate for special education teachers is probably higher than that for other teachers (Billingsley, 1993), resulting in insufficient numbers of special education teachers (Billingsley and Bettini, 2019). Consequently, the shortage of special educators implies that the needs of special education development cannot be met. One of the key reasons is that special education teachers are under pressure, and their work engagement and well-being are relatively low (Edward et al., 2018); thus, it is critical to improve their engagement and occupational well-being.

The job demands-resources (JD-R) model suggests that work-related personal resources [e.g., psychological capital (PsyCap)] contribute to generating greater work engagement (Demerouti et al., 2001) and have a positive influence on work outcomes (Xanthopoulou et al., 2007), including workers’ well-being (Wang et al., 2017). Similarly, the conservation of resources (COR) theory posits that workers with more resources (such as PsyCap) are likely to engage more deeply in work and generate better outcomes (Hobfoll, 2002). People who engage more deeply with work may experience greater subjective well-being (Caesens et al., 2014), and empirical research has also shown that resource support can improve special education teachers’ working conditions (Ghere and York-Barr, 2007; Dempsey and Christenson-Foggett, 2011).

Could PsyCap be used as a positive resource to predict well-being through work engagement? Thus far, only one study has explored this question; Adil and Kamal (2016) found that work engagement might mediate the association between university teachers’ PsyCap and their job-related well-being. As special education teachers work with students with special needs, their professional characteristics differ from those of other teachers (Laloo and Buhril, 2013). Whether the mediating effects of work engagement is applicable for special education teachers? Furthermore, Adil and Kamal (2016) do not explain the specific effects of the three dimensions of work engagement in the association between PsyCap and occupational well-being. Therefore, our research attempts to verify the indirect effect of PsyCap on occupational well-being through work engagement and further explore the mediating role of the three dimensions of work engagement in the association between PsyCap and occupational well-being. To the best of our knowledge, the current study is the first to explore these issues among Chinese special education teachers specifically. Our findings will help education managers and policymakers understand how to improve special education teachers’ PsyCap, which will, in turn, improve work engagement and enhance their occupational well-being, resulting in decreased turnover.

## Occupational Well-Being

Occupational well-being relates to an individual’s experience and satisfaction at work (Doble and Santha, 2008). Huang (2014) suggested that occupational well-being is the quality of a worker’s experience and effectiveness at work. However, special education teachers experience less well-being in their work (Ouellette et al., 2019; Soini et al., 2019). Children with special needs are individually diverse and require a high level of competence from special education teachers, yet their development is extremely slow. This leads to higher levels of work stress and poor mental health among special education teachers (He, 2016; Poppes et al., 2016; Sun et al., 2019), even resulting in higher job burnout and lower well-being (Zhang and Zhang, 2012; Black and Halstead, 2021). Previous studies have confirmed that the occupational well-being of special education teachers was lower than that of mainstream school teachers in China (Zhao and Huang, 2015). To address this problem, some researchers have explored the protective factors of special education teachers’ occupational well-being (Wu et al., 2020; Xu et al., 2021), such as PsyCap, work engagement and so on (Wang, 2017; Wen and Zhang, 2020).

## Psychological Capital and Occupational Well-Being

Psychological capital is an active psychological state, comprising optimism, self-efficacy, resilience, and hope (Luthans et al., 2007b). Empirical research has shown that PsyCap as a whole predicts better outcome variables than each of its individual facets (Luthans et al., 2007a). According to the JD-R model, individual resources (e.g., PsyCap) lead to work engagement and, consequently, bring about positive outcomes, such as personal well-being and professional identity (Xanthopoulou et al., 2007). This may be because people with high levels of PsyCap make greater efforts to overcome challenging tasks and adversity, possess positive attributes and firm goals, and ultimately attain success (Luthans et al., 2007b). That could be able to help workers experience improved well-being (Avey et al., 2011), and, in turn, their turnover intentions may reduce (Siu et al., 2015). Kanapathy et al. (2016) found that PsyCap positively predicts special education teachers’ subjective well-being. Further, Peng et al. (2018) conducted a survey of special education teachers in southwest China (Chongqing and Sichuan province) and demonstrated that all dimensions of PsyCap—optimism, self-confidence, resilience, and hope—are positively related to occupational happiness. Together, PsyCap could be a one of protective factors of special education teachers’ well-being.

## Work Engagement and Occupational Well-Being

Work engagement describes a fulfilling and energetic work-related mental state and comprises three dimensions: absorption, dedication, and vigor (Schaufeli et al., 2006). Dedication means engagement and enthusiasm regarding work and its challenges, and the perception that work is important. Absorption means that workers entirely concentrate on and become engrossed in their work—time quickly passes—and they are unwilling to break away. Finally, vigor means that an individual has sufficient energy and psychological resilience, and can persist when they experience difficulties (Schaufeli et al., 2002). According to the JD-R model, researchers have assumed that work engagement is a motivational construct that can improve performance at work (Schaufeli and Bakker, 2004). Individuals engaging in work often experience positive emotions, such as enthusiasm and well-being (Yang et al., 2019). For example, Wang (2017) contends that special education teachers with high levels of work engagement experience positive well-being. An empirical study has shown that special education teachers’ work engagement is a positive predictor of general well-being (Fu et al., 2021).

## Mediation of Work Engagement

Bakker and Demerouti (2008) assert that individual resources (e.g., PsyCap), functioning as motivational processes, can predict work engagement. Consistent with this view, COR theory proposes that the higher the PsyCap level, the greater the motivation a worker experiences, and this encourages them to focus on work (Sweetman and Luthans, 2010). Previous studies have found the importance of PsyCap in promoting work engagement (Xanthopoulou et al., 2009; Kang and Busser, 2018). For example, Jin (2017) investigated 253 front-line special education teachers and found that those with high professional identification had higher PsyCap and were more actively focused on their work. Thus, we propose that PsyCap can facilitate special education teachers’ work engagement. Furthermore, in accordance with COR theory, and considering the association between PsyCap, work engagement, and occupational well-being, we expect that special education teachers with high PsyCap experience better engagement with their work, leading to higher levels of occupational well-being.

Although some scholars have asserted that the scores for the three dimensions of work engagement should be equally used in empirical research, other studies have shown that they play different roles in the mediation model (Matthews et al., 2014; Scrima et al., 2014; Van Bogaert et al., 2014). Matthews et al. (2014) demonstrated that work engagement acts as a mediator in the association between family-supportive supervisor behaviors (FSSBs) and subjective well-being. However, when the three components were set in a multiple mediation model to test the relationship between FSSBs and subjective well-being, the results indicated that only vigor significantly mediated the relationship. Thus, to understand how PsyCap predicts occupational well-being, it is necessary to explore its relationships with the three dimensions.

## The Present Study

Although some researchers have explored the relationship between PsyCap, work engagement, and well-being, the mediating role of work engagement has not been explored among special education teachers. The current study offers insight into the relationship between PsyCap, work engagement, and occupational well-being in the Chinese context. Consistent with the JD-R model and COR theory, we hypothesized that:

H1. PsyCap has a significantly positive direct effect on work engagement and occupational well-being for special education teachers.

H2. PsyCap of special education teachers might indirectly predict occupational well-being through work engagement.

Additionally, considering previous empirical evidence regarding the mediation effect of the dimensions of work engagement, we hypothesized that:

H3. Dedication, absorption, and vigor might be correlated with PsyCap and occupational well-being, but not all of them necessarily mediate the relationship between PsyCap and occupational well-being.

## Materials and Methods

### Participants and Data Collection

The random cluster sampling method was adopted in this study, and samples were drawn from special education teachers from five Chinese provinces (Shandong, Jiangsu, Zhejiang, Anhui, and Fujian) and the city of Shanghai. The purpose and details of the study were provided to all the participants. After written consent, they were invited to complete an anonymous survey consisting of demographic items and questions. Completion of the survey took 15–20 min. The study was approved by the Human Research Ethics Committee of (Zhejiang Normal University).

A total of 650 questionnaires were distributed to the participants, and the return rate was 96.15%. Some questionnaire data were excluded due to the regularity of responses to questions. Among the 625 questionnaires, 615 were found valid, with an effective rate of 94.62%. More than 92% of the participants were female *(n* = 567), and 8% were male (*n* = 48). Most participants (68.6%) reported less than 10 years of work experience as special education teachers, while 17.4% had worked in the role for more than 21 years. Approximately 64.6% of the participants had undergraduate degrees, and 8.8% had master’s degrees. Finally, 7.3% of the participants reported an average monthly income below 2500 RMB ($375.60), 27.6% earned between 2501 and 4000 RMB ($375.75–$600.95), 37.4% reported between 4001 and 5500 RMB ($601.10–$826.31), and 27.6% earned more than 5500 RMB ($826.46).

### Measures

#### Psychological Capital Questionnaire

The 24-item revised Chinese version of the Psychological Capital Questionnaire (Li, 2007) was used to evaluate special education teachers’ PsyCap. This version includes four dimensions—optimism, self-efficacy, resilience, and hope—and has been demonstrated to have good reliability and validity. The participants responded to all the items on a 6-point Likert response scale (1 = strongly disagree, 6 = strongly agree). In the present study, Cronbach’s α of the scale was 0.94.

#### Utrecht Work Engagement Scale

The revised Chinese version of the Utrecht Work Engagement Scale was used to assess work engagement, which has been demonstrated to have good reliability and validity (Zhang and Gan, 2005). This questionnaire includes 17 items rated on a 7-point Likert response scale (0 = never, 6 = always) and comprises three dimensions—dedication, absorption, and vigor. Higher scores signify better work engagement. In the present study, the Cronbach’s α of subscales of dedication, absorption, and vigor were 0.84, 0.90, and 0.92, respectively. The Cronbach’s α of the scale was 0.95.

#### Special Education Teachers’ Occupational Well-Being Questionnaire

Occupational well-being was measured using the Special Education Teachers’ Occupational Well-Being Questionnaire (SETOWQ; Wang, 2017), which includes 25 items that assess occupational well-being in five dimensions—emotional happiness, professional happiness, physical and mental pleasure, environmental satisfaction, and interpersonal harmony. All items are measured on a 5-point Likert response scale (1 = strongly disagree, 5 = strongly agree), and this scale has been confirmed to be valid and reliable (Wang, 2017). In the present study, the Cronbach’s α of the scale was 0.93.

### Data Analysis

Descriptive statistics and correlation analyses were performed using SPSS20.0 and Mplus7.4 to explore the predictive effect of PsyCap as an independent variable on occupational well-being, and the mediation effects of work engagement in the association between PsyCap and occupational well-being. Harman’s single-factor test was used to examine the common method biases that may arise from self-reporting in cross-sectional studies. Exploratory factor analysis of all the items revealed that the first factor of the total variance was 20.86%, which was less than the critical value of 40%. Therefore, no common method variance was found in this study’s data.

## Results

### Descriptive Analyses

Table 1 presents the means (M), standard deviations (SD), and correlations of all the variables. As expected, PsyCap was positively associated with occupational well-being (*r* = 0.60, *p* < 0.01) and work engagement (*r* = 0.55, *p* < 0.01). Work engagement was positively associated with occupational well-being (*r* = 0.53, *p* < 0.01), while dedication, absorption, and vigor were positively associated with PsyCap (*r* = 0.53, 0.47, and 0.50, respectively, *p* < 0.01) and occupational well-being (*r* = 0.55, 0.46, and 0.45, respectively, *p* < 0.01).

**TABLE 1:** Mean, Standard Deviations, and Correlations Between the Measures (*N* = 615).

	*M*	SD	1	2	3	4	5	6
1. PsyCap	4.70	0.52	–					
2. WE	4.25	0.78	0.55**	–				
3. Dedication	4.78	0.84	0.53**	0.90**	–			
4. Vigor	4.27	0.83	0.50**	0.92**	0.77**	–		
5. Absorption	3.79	0.89	0.47**	0.91**	0.71**	0.75**	–	
6. OWB	4.07	0.48	0.60**	0.53**	0.55**	0.45**	0.46**	–

PsyCap, psychological capital; WE, work engagement; OWB, occupational well-being. **p < 0.01.

### Measurement Model

According to Anderson and Gerbing (1988), the measurement model of all latent variables and manifest variables should be tested before examining the structural model. The hypothesized measurement model in the current study included three interrelated latent variables—PsyCap, work engagement, and occupational well-being—as well as 13 manifest variables. The confirmatory factor analysis indicated that the measurement model had an acceptable fit to the data: χ^2^ = 237.93, *p* < 0.001, CFI = 0.95, TLI = 0.94, RMSEA = 0.07 [90% CI = (0.07, 0.09)], SRMR = 0.05.

### Structural Model

**FIGURE 1:**
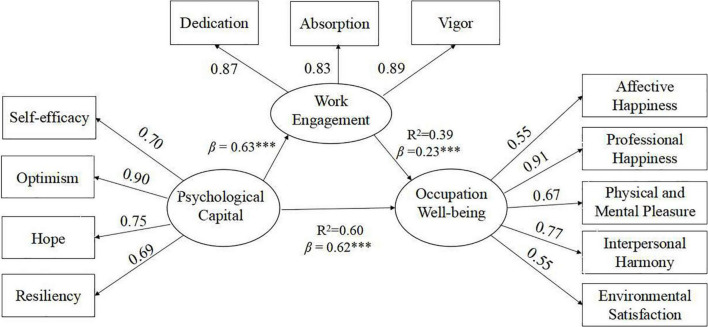
Standardized path estimates for the latent structural models (*N* = 615, ***p < 0.001).

Our main assumption was that PsyCap has a significantly positive direct effect on special education teachers’ occupational well-being and work engagement (H1). We hypothesized that special education teachers’ work engagement mediates the association between PsyCap and occupational well-being (H2). We examined these hypotheses using structural equation modeling. The hypothesized model indicated an acceptable fit: χ^2^ = 411.11, RMSEA = 0.07 [90% CI = (0.07, 0.08)], CFI = 0.93, TLI = 0.91, SRMR = 0.05. The results showed that special education teachers’ PsyCap had significantly positive direct effects on their occupational well-being (β = 0.62, *p* < 0.001) and work engagement (β = 0.63, *p* < 0.001), confirming Hypothesis 1 (see Figure 1). To examine Hypothesis 2, we used the maximum likelihood bootstrap estimation procedure in Mplus7.4. All relevant effects were estimated with standard errors and confidence intervals (95%; 2,000 samples were drawn). The results indicated PsyCap had indirect effects on special education teachers’ occupational well-being *via* work engagement. The effects and associated 95% CI are shown in Table 2.

**TABLE 2:** The Effects and Associated 95% CI for the Mediation Model 1.

Model pathways	Estimated	*SE*	95% CI	
			LL	UL
PsyCap → WE → OWB	0.14	0.03	0.08	0.20
PsyCap → OWB	0.62	0.05	0.53	0.73

*SE, standard error; CI, confidence interval; LL, lower limit; UL, upper limit; PsyCap, psychological capital; WE, work engagement; OWB, occupational well-being.*

**FIGURE 2:**
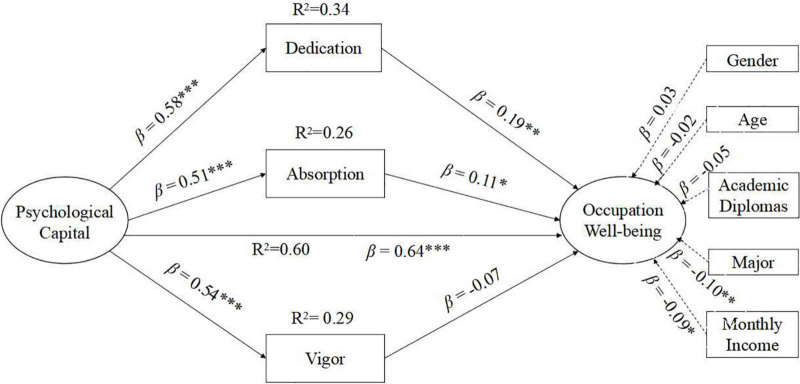
Standardized path estimates for the structural models (*p < 0.05, **p < 0.01, ***p < 0.001).

As work engagement contains three dimensions (absorption, dedication, and vigor), we conducted a multiple mediation analysis (Preacher and Hayes, 2008) to examine the mediational contribution of the components (H3). Dedication, absorption, and vigor were included as mediators between PsyCap and occupational well-being, and demographic variables were treated as covariates (gender, age, academic diplomas, major, and monthly income). The fit of the multiple mediation model was found acceptable: χ^2^ = 336.76, *p* < 0.001, RMSEA = 0.07 [90% CI = (0.06, 0.08)], CFI = 0.94, TLI = 0.90 SRMR = 0.04. As presented in the model (Figure 2), PsyCap positively predicted dedication (β = 0.58, *p* < 0.001), absorption (β = 0.51, *p* < 0.001), vigor (β = 0.54, *p* < 0.001), and occupational well-being (β = 0.64, *p* < 0.001). The indirect effects of PsyCap on occupational well-being *via* dedication and absorption were significant and positive (indirect effect = 0.11, 0.06), and the 95% bias-corrected bootstrap confidence interval did not contain 0 [95% CI = (0.05, 0.17), (0.01, 0.11)], except for vigor [95% CI = (−0.10, 0.02)].

Further examinations were conducted to test the size of the specific indirect effects. The results showed no significant difference between the mediating effects produced by dedication and absorption (*Z* = 1.23, *p* < 0.05), and the 95% confidence interval included 0 [95% CI = (−0.03, 0.13)]. The effect produced *via* vigor significantly differed from those *via* dedication (*Z* = 2.83, *p* < 0.01) and absorption (*Z* = 2.01, *p* < 0.05), and the 95% confidence interval did not include 0 [95% CI = (0.04, 0.22), 95% CI = (0.01, 0.15)]. Therefore, dedication and absorption can be considered as the primary mediators of the relationship between PsyCap and occupational well-being.

## Discussion

This study explored the association between PsyCap, work engagement, and occupational well-being of special education teachers and investigated the mediating roles of work engagement and its dimensions in the association between PsyCap and occupational well-being. We found that PsyCap of Chinese special education teachers had a significantly positive direct effect on occupational well-being, which corroborates previous evidence that PsyCap helps promote workers’ performance and well-being (Kanapathy et al., 2016). COR theory argues that people strive to acquire and maintain personal resources, such as PsyCap (Hobfoll, 1989), which may contribute to the achievement of goals and improvement of well-being (Howard, 2018; Newman et al., 2018). PsyCap not only improves primary school teachers’ well-being but also reduces their work stress levels, ultimately leading to a greater sense of well-being (Zhao and Zhang, 2014). Peng et al. (2018) deem that special education teachers with greater PsyCap are better at dealing with problems at work and have a greater sense of occupational well-being. Furthermore, the effect of PsyCap on well-being persists over time, and positive personal resources aid teachers in finding more meaning in life, which in turn enhances their well-being (Li, 2018).

Consistent with the JD–R model, our results suggested that PsyCap positively predicted special education teachers’ work engagement, which supports prior findings (Khaleghkhah et al., 2017; Li et al., 2018). The JD-R model proposes that the characteristics of work can be divided into two types, job resources and job demands (Demerouti et al., 2001). Job demands consume individual resources and can consequently lead to burnout, while job resources aid individuals in reducing personal consumption, reaching goals, and promoting individual development (Bakker and Demerouti, 2007). PsyCap is a personal resource that can provide teachers with energy, stimulate motivation, and promote work engagement (Mao and Xie, 2013). Special education teachers who believe in their abilities and have a positive view of engagement in work will perform well in their jobs, even when faced with difficulties; thus, they will be able to continue with their duties and remain fully engrossed in their work (Peng et al., 2018).

It is noteworthy that, in this study, a positive relationship between work engagement and occupational well-being was observed, and this relationship mediated the association between PsyCap and occupational well-being of the Chinese special education teachers. This finding is consistent with that of Adil and Kamal’s (2016) study of university teachers in Pakistan. Their results indicated that higher levels of PsyCap among special education teachers enhance their work engagement, which in turn increases their occupational well-being.

To better reveal the internal mechanisms of PsyCap and occupational well-being, the current study conducted a multiple mediation analysis, setting the dimensions of work engagement (dedication, vigor, and absorption) as mediators. The results suggested that dedication and absorption mediated the association between PsyCap and occupational well-being, indicating that the role of those dimensions was not equal in the mediation relationship model. These results are consistent with those of previous research (Matthews et al., 2014) and may be due to the following reasons.

First, the findings may be related to the special education teaching profession. Most special education teachers tend to be caring and aware of the value and significance of their work with children with special needs, their families, and society. Therefore, special education teachers tend to focus on their work and actively participate in teaching and learning activities. They grow and gain happiness from their work to some extent.

Second, the vigor level of special education teachers is low. Although Chinese special education teachers often draw on resources of love and patience, their work is often considered trivial, monotonous, and repetitive, differing from the work of teachers involved with imparting scientific and cultural knowledge. In addition, primary school teachers usually have clear goals and are motivated to help their pupils achieve the best results that can enable them to gain access to good high schools. However, in special education, such goals do not exist, and there is little competition for promotion, so there is no sense of urgency that stimulates vigor (Chen et al., 2010). Furthermore, children with special educational needs often have functional or physiological disorders, and their teachers must practice stratified teaching and individualized rehabilitation according to each child’s needs. The developmental progress of children with special needs is often slow. In addition, Chinese special education teachers demonstrate less competency in these occupational skills. Therefore, teachers’ psychological resilience and vigor are easily eroded, resulting in lower work-related happiness (Chen and Yang, 2017), and even burnout (Garwood et al., 2018). Therefore, PsyCap could not predict occupational well-being through vigor.

## Implications

In the current study, a positive association between PsyCap, work engagement, and occupational well-being of Chinese special education teachers validated the JD-R model and COR theory. Our findings imply that PsyCap is positively correlated with work engagement and occupational well-being; that is, teachers with higher PsyCap are more likely to experience work engagement and feel happier in Chinese special education schools. In line with our expectations, the association between PsyCap and occupational well-being was partially mediated by work engagement, implying that the PsyCap of special education teachers can predict their work engagement, which, in turn, can enhance their well-being. Specifically, the indirect effect of PsyCap on occupational well-being mainly occurs *via* dedication and absorption.

Some insights can be drawn from the results. Special education schools should guide teachers to pay attention to their PsyCap and help them learn how to self-regulate to improve their PsyCap levels. For example, their teaching skills can be improved through effective teaching demonstrations and group seminars so that their sense of efficacy is enhanced. In addition, schools can create a participative working atmosphere and encourage a harmonious professional relationship among teachers. This will promote teachers’ positive emotional experience and encourage them to better devote themselves to their work, improving their occupational well-being.

To the best of our knowledge, this is the first study to explore the mediating role of work engagement in the association between PsyCap and occupational well-being in the Chinese context. The study was based on the JD-R model and COR theory and applied them to the domain of Chinese special education. Thereby, the study contributes to the empirical literature and aids further exploration of possible methods to improve special education teachers’ enthusiasm for their work. The partial indirect effect of PsyCap on occupational well-being indicates that there might be other variables affecting this relationship, such as teacher efficacy and social support (Dixon et al., 2014). Future research could examine external resources and other personal resources in this model.

## Limitations

Although this study’s results contribute to the understanding of the internal mechanisms of how work engagement operates in the relationship between PsyCap and occupational well-being among Chinese special education teachers, several limitations should be noted. First, this study adopted a cross-sectional design; thus, it is difficult to confirm causal relationships between the variables. Future studies could test these results using longitudinal studies to determine the causal relations among PsyCap, work engagement, and occupational well-being. Second, collection of PsyCap, work engagement, and occupational well-being data depended on self-report questionnaires, which could have been influenced by personal subjectivity to some extent. In the future, researchers may consider using qualitative and quantitative methods. Third, this study’s participants were mainly sampled from the east coast of China; therefore, the results cannot be generalized to special education teachers across the whole country. Consequently, future studies should expand the sample size to examine the applicability of our results to the general population.

## Conclusion

This study provides insights regarding the association between PsyCap, work engagement, and occupational well-being of Chinese special education teachers. Our research demonstrated that PsyCap and work engagement are positively associated with occupational well-being and that Chinese special education teachers’ work engagement can mediate the association between their PsyCap and occupational well-being. Notably, we found that work engagement plays an important role through which PsyCap enriches teachers’ occupational well-being. Specifically, dedication and absorption were found to be the primary mediators of PsyCap and occupational well-being.

## Data Availability Statement

The raw data from this study are relevant to subsequent research and therefore unavailable. If researchers are interested in or need the data, they can contact the first author of this study.

## Ethics Statement

The studies involving human participants were reviewed and approved by the Human Ethics Committee of Zhejiang Normal University. The participants signed the informed consent form and completed the questionnaire anonymously.

## Author Contributions

QG designed this study, collected the data, and wrote and revised the manuscript. YW wrote and revised the manuscript. QL and TW revised the manuscript. LZ collected the data. ZH wrote the manuscript. SC designed the study and revised the manuscript. All authors contributed to the article and approved the submitted version.

## Conflict of Interest

The authors declare that the research was conducted in the absence of any commercial or financial relationships that could be construed as a potential conflict of interest.

## Publisher’s Note

All claims expressed in this article are solely those of the authors and do not necessarily represent those of their affiliated organizations, or those of the publisher, the editors and the reviewers. Any product that may be evaluated in this article, or claim that may be made by its manufacturer, is not guaranteed or endorsed by the publisher.

## References

[B1] Adil A. Kamal A. (2016). Impact of psychological capital and authentic leadership on work engagement and job related affective well-being. *Pakistan J. Psychol. Res.* 31 1–21.

[B2] Anderson J. Gerbing D. (1988). Structural equation modeling in practice: A review of recommended two-step approach. *Psychol. Bull.* 103 411–423. 10.1037/0033-2909.103.3.411

[B3] Avey J. B. Reichard R. J. Luthans F. Mhatre K. H. (2011). Meta-analysis of the impact of positive psychological capital on employee attitudes, behaviors, and performance. *Hum. Res. Dev. Q.* 22 127–152. 10.1002/hrdq.20070

[B4] Bakker A. B. Demerouti E. (2007). The Job Demands-Resources model: state of the art. *J. Manage. Psychol.* 22 309–328. 10.1108/02683940710733115

[B5] Bakker A. B. Demerouti E. (2008). Towards a model of work engagement. *Career Dev. Int.* 13 209–223. 10.1108/13620430810870476

[B6] Billingsley B. Bettini E. (2019). Special education teacher attrition and retention: A review of the literature. *Rev. Educ. Res.* 89 697–744. 10.3102/0034654319862495

[B7] Billingsley B. S. (1993). Teacher retention and attrition in special and general education: A critical review of the literature. *J. Special Educ.* 27 137–174. 10.1177/002246699302700202

[B8] Billingsley B. S. (2004). Special education teacher retention and attrition: A critical analysis of the research literature. *J. Special Educ.* 38 39–55. 10.1177/00224669040380010401

[B9] Black N. Halstead E. (2021). Mental health and subjective well-being of staff in a secondary school for adolescents with severe and profound multiple learning difficulties. *Br. J. Special Educ.* 48 477–496. 10.1111/1467-8578.12389

[B10] Caesens G. Stinglhamber F. Luypaert G. (2014). The impact of work engagement and workaholism on well-being: The role of work-related social support. *Career Dev. Int.* 19 813–835. 10.1108/CDI-09-2013-0114

[B11] Chen L. Fu N. Wang R. (2010). The predictive effect of work resources on work engagement of special education teachers. *Chin. J. Special Educ.* 1 48–54.

[B12] Chen L. Yang J. (2017). Relationship between special education teachers’ professional identity, turnover intention and job satisfaction. *Chin. J. Special Educ.* 2 25–30.

[B13] Demerouti E. Bakker A. B. Nachreiner F. Schaufeli W. B. (2001). The job demands-resources model of burnout. *J. Appl. Psychol.* 86 499–512. 10.1037//0021-9010.86.3.499 11419809

[B14] Dempsey I. Christenson-Foggett J. (2011). External mentoring support for early career special education teachers. *Aust. J. Special Educ.* 35 61–71. 10.1375/ajse.35.1.61

[B15] Dixon D. Yssel N. McConnell J. M. Hardin T. (2014). Differentiated instruction, professional development, and teacher efficacy. *J. Educ. Gift.* 37 111–127. 10.1177/0162353214529042

[B16] Doble S. E. Santha J. C. (2008). Occupational well-being: rethinking occupational therapy outcomes. *Can. J. Occup. Ther.* 75 184–190. 10.1177/000841740807500310 18615930

[B17] Edward C. Larsen R. Mathur S. Estes M. Johns B. Chang M. (2018). Special education teacher Stress: Coping strategies. *Educ. Treat. Child.* 41 457–481. 10.1353/etc.2018.0025 34409987

[B18] Fu W. Wang C. Tang W. Lu S. Wang Y. (2021). Emotional intelligence and well-being of special education teachers in China: The mediating role of work engagement. *Front. Psychol.* 12:696561. 10.3389/fpsyg.2021.696561 34526933 PMC8435597

[B19] Garwood J. D. Werts M. G. Varghese C. Gosey L. (2018). Mixed-methods analysis of rural special educators’ role stressors, behavior management, and burnout. *Rural Special Educ. Q.* 37 30–43. 10.1177/8756870517745270

[B20] Ghere G. York-Barr J. (2007). Paraprofessional turnover and retention in inclusive programs: Hidden costs and promising practices. *Remed. Special Educ.* 28 21–32. 10.1177/07419325070280010301

[B21] He Y. (2016). A study of subjective well-being, working pressure and relative of special education school teachers. *J. Modern Special Educ.* 8, 57–62.

[B22] Hobfoll S. E. (1989). Conservation of resources: A new attempt at conceptualizing stress. *Am. Psychol.* 44 513–524. 10.1037/0003-066X.44.3.513 2648906

[B23] Hobfoll S. E. (2002). Social and psychological resources and adaptation. *Rev. Gen. Psychol.* 6 307–324. 10.1037//1089-2680.6.4.307

[B24] Howard M. C. (2018). The measurement, nomological net, and theory of perceived self-esteem instability: Applying the conservation of resources theory to understand the construct. *Psychol. Rep.* 122 1007–1042. 10.1177/0033294118781319 29871532

[B25] Huang L. (2014). On the dimensional structure of the employee occupational well-being in Chinese enterprises. *J. Central University Finance Econ.* 10 84–92.

[B26] Jin Q. (2017). *The Research on a Relationship among Special Education teachers’ professional identity, psychological capital and job involvement.* (Ph.D thesis), China: University of Jinan.

[B27] Kanapathy R. Majid R. A. Amat S. Yasin M. H. M. (2016). Relationship and contribution of psychological capital and organizational support towards the subjective well-being of special education secondary school teachers in the Central Region of Peninsula Malaysia. *Asia Pacific J. Intell. Disabil.* 3 12–19.

[B28] Kang H. J. A. Busser J. A. (2018). Impact of service climate and psychological capital on employee engagement: The role of organizational hierarchy. *Int. J. Hospital. Manage.* 75 1–9. 10.1016/j.ijhm.2018.03.003

[B29] Khaleghkhah A. Babelan A. Z. Karimianpour G. (2017). Prediction of job engagement of teachers based on psychological capital and psychological hardiness. *Revista La Universidad Del Zulia* 8 33–47.

[B30] Laloo B. Buhril J. L. (2013). Information needs and information seeking behavior of teachers of special education in Shillong, India. *J. Am. Acad. Special Educ. Profession.* 48:69.

[B31] Li C. P. (2007). *Psychological Capital.* Beijing: China Light Industry Press.

[B32] Li Y. (2018). Building well-being among university teachers: the roles of psychological capital and meaning in life. *Eur. J. Work Organ. Psychol.* 27 594–602. 10.1080/1359432X.2018.1496909

[B33] Li Y. Z. Castano G. Li Y. (2018). Linking leadership styles to work engagement: The role of psychological capital. *Chin. Manage. Stud.* 12 433–452. 10.1108/CMS-04-2017-0108

[B34] Luthans F. Youssef C. M. Avolio B. J. (2007b). *Psychological Capital: Developing the Human Competitive Edge.* Oxford, UK: Oxford University Press.

[B35] Luthans F. Avolio B. J. Avey J. B. Norman S. M. (2007a). Positive psychological capital: Measurement and relationship with performance and satisfaction. *Person. Psychol.* 60 541–572. 10.1111/j.1744-6570.2007.00083.x

[B36] Mao J. P. Xie Y. (2013). A positive study of lower school teachers’ psychological capital and work involvement. *Teach. Educ. Res.* 25 23–29.

[B37] Matthews R. A. Mills M. J. Trout R. C. English L. (2014). Family-supportive supervisor behaviors, work engagement, and subjective well-being: A contextually dependent mediated process. *J. Occup. Health Psychol.* 19 168–181. 10.1037/a0036012 24730426

[B38] Newman A. Nielsen I. Smyth R. Hirst G. (2018). Mediating role of psychological capital in the relationship between social support and wellbeing of refugees. *Int. Migrat.* 56 117–132. 10.1111/imig.12415

[B39] Ouellette R. R. Pellecchia M. Beidas R. S. Wideman R. Xie M. Mandell D. S. (2019). Boon or burden: The effect of implementing evidence-based practices on teachers’ emotional exhaustion. *Admin. Policy Ment. Health Res.* 46 62–70. 10.1007/s10488-018-0894-6 30225662 PMC6400069

[B40] Peng O. Huang X. Wang G. Zhang R. N. Bai W. (2018). The effect of special education teachers’ competency on their occupational well-being: the mediating effect of psychological capital. *Chin. J. Special Educ.* 10 51–55.

[B41] Poppes P. Putten A. Post W. Frans N. Brug A. Es A. (2016). Relabelling behaviour. The effects of psycho-education on the perceived severity and causes of challenging behaviour in people with profound intellectual and multiple disabilities: Relabelling behaviour. *J. Intell. Disabil. Res.* 60 1140–1152. 10.1111/jir.12299 27189898

[B42] Preacher K. J. Hayes A. F. (2008). Asymptotic and resampling strategies for assessing and comparing indirect effects in multiple mediator models. *Behav. Res. Methods* 40 879–891. 10.3758/BRM.40.3.879 18697684

[B43] Schaufeli W. Salanova M. González-romá V. Bakker A. (2002). The measurement of engagement and burnout: A two sample confirmatory factor analytic approach. *J. Happ. Stud.* 3 71–92. 10.1023/A:1015630930326

[B44] Schaufeli W. B. Bakker A. B. (2004). Job demands, job resources, and their relationship with burnout and engagement: a multi-sample study. *J. Organ. Behav.* 25 293–315. 10.1002/job.248

[B45] Schaufeli W. B. Bakker A. B. Salanova M. (2006). The measurement of work engagement with a short questionnaire: a cross-national study. *Educ. Psychol. Measure.* 66 701–716. 10.1177/0013164405282471

[B46] Scrima F. Lorito L. Parry E. Falgares G. (2014). The mediating role of work engagement on the relationship between job involvement and affective commitment. *Int. J. Hum. Res. Manage.* 25 2159–2173. 10.1080/09585192.2013.862289

[B47] Siu O. L. Cheung F. Lui S. (2015). Linking positive emotions to work well-being and turnover intention among Hong Kong police officers: The role of psychological capital. *J. Happiness Stud.* 16 367–380. 10.1007/s10902-014-9513-8

[B48] Soini T. Pietarinen J. Pyhältö K. Haverinen K. Jindal Snape D. Kontu E. (2019). Special education teachers’ experienced burnout and perceived fit with the professional community: A 5-year follow-up study. *Br. Educ. Res. J.* 45 622–639. 10.1002/berj.3516

[B49] Sun J. Wang Y. Wan Q. Huang Z. (2019). Mindfulness and special education teachers’ burnout: The serial multiple mediation effects of self-acceptance and perceived stress. *Soc. Behav. Personal.* 47:e8656. 10.2224/sbp.8656

[B50] Sweetman D. Luthans F. (2010). “The power of positive psychology: Psychological capital and work engagement”. in *Work engagement* (1st ed.) Eds Bakker A. Leiter M (New York, NY: Psychology Press), 54–68. 10.4324/9780203853047

[B51] Van Bogaert P. van Heusden D. Timmermans O. Franck E. (2014). Nurse work engagement impacts job outcome and nurse-assessed quality of care: model testing with nurse practice environment and nurse work characteristics as predictors. *Front. Psychol.* 5:1261. 10.3389/fpsyg.2014.01261 25431563 PMC4230203

[B52] Wang G. Huang X. Zhang D. J. (2017). Effects of occupational stress and psychological capital on occupational well-being among kindergarten teachers: effects of coping way and culture. *Stud. Psychol. Behav.* 15 83–91. 10.13718/j.cnki.xdzk.2014.10.027

[B53] Wang X. (2017). *Professional identity, professional well-being and their relationship with job involvement of special education teachers.* (Ph.D thesis). Maharashtra: Southwest University.

[B54] Wen Y. Zhang S. (2020). The relationship between emotional labor and occupational well-being of special education teachers: mediating role of psychological capital. *J. Modern Special Educ.* 14, 19–25.

[B55] Wu T. Wang L. Gao J. Wei A. (2020). Social support and well-being of Chinese special education teachers-An emotional labor perspective. *Int. J. Env. Res. Public Health* 17:6884. 10.3390/ijerph17186884 32967136 PMC7558049

[B56] Xanthopoulou D. Bakker A. B. Demerouti E. Schaufeli W. B. (2007). The role of personal resources in the job demands-resources model. *Int. J. Stress Manage.* 14 121–141. 10.1037/1072-5245.14.2.121

[B57] Xanthopoulou D. Bakker A. B. Demerouti E. Schaufeli W. B. (2009). Reciprocal relationships between job resources, personal resources, and work engagement. *J. Vocat. Behav.* 74 235–244. 10.1016/j.jvb.2008.11.003

[B58] Xu N. Chen P. Lang R. Kong L. Qu H. (2021). The effect of Chinese special education teachers’ competence on their occupational well-being: The mediating effect of resilience. *Int. J. Disabil. Dev. Educ.* 2021 1–16. 10.1080/1034912X.2021.1975263

[B59] Yang X. Feng Y. Meng Y. Qiu Y. (2019). Career adaptability, work engagement, and employee well-being among chinese employees: The role of guanxi. *Front. Psychol.* 10:1029. 10.3389/fpsyg.2019.01029 31139112 PMC6527591

[B60] Zhang Y. Zhang Y. (2012). The report of the special teachers situation in Sichuan. *J. School. Stud.* 9 92–98.

[B61] Zhang Y. W. Gan Y. Q. (2005). The Chinese version of Utrecht Work Engagement Scale: an examination of reliability and validity. *Chin. J. Clin. Psychol.* 13 268–270. 10.16128/j.cnki.1005-3611.2005.03.005

[B62] Zhao B. Huang Y. (2015). Comparative study of occupational well-being of teachers from special education schools and regular schools. *J. Modern Special Educ.* 5 56–61.

[B63] Zhao B. Zhang D. J. (2014). Research on the relationship between teachers’ work related well being and work engagement. *Modern Primary Second. Educ.* 30 108–111.

